# Effect of Physical Exercise on the Febrigenic Signaling is Modulated by Preoptic Hydrogen Sulfide Production

**DOI:** 10.1371/journal.pone.0170468

**Published:** 2017-01-24

**Authors:** Jonatas E. Nogueira, Renato N. Soriano, Rodrigo A. R. Fernandez, Heloísa D. C. Francescato, Rafael S. Saia, Terezila M. Coimbra, José Antunes-Rodrigues, Luiz G. S. Branco

**Affiliations:** 1 Postgraduate Program in Rehabilitation and Functional Performance, University of São Paulo, Ribeirão Preto, SP, Brazil; 2 School of Physical Education and Sports of Ribeirao Preto, University of São Paulo, Ribeirão Preto, SP, Brazil; 3 Federal University of Juiz de Fora, Governador Valadares, MG, Brazil; 4 Department of Physiology, School of Medicine of Ribeirão Preto, University of São Paulo, Ribeirão Preto, SP, Brazil; 5 Department of Morphology, Physiology, and Basic Pathology, Dental School of Ribeirão Preto, University of São Paulo, Ribeirão Preto, SP, Brazil; St. Joseph's Hospital and Medical Center, UNITED STATES

## Abstract

We tested the hypothesis that the neuromodulator hydrogen sulfide (H_2_S) in the preoptic area (POA) of the hypothalamus modulates the febrigenic signaling differently in sedentary and trained rats. Besides H_2_S production rate and protein expressions of H_2_S-related synthases cystathionine β-synthase (CBS), 3-mercaptopyruvate sulfurtransferase (3-MPST) and cystathionine γ-lyase (CSE) in the POA, we also measured deep body temperature (Tb), circulating plasma levels of cytokines and corticosterone in an animal model of systemic inflammation. Rats run on a treadmill before receiving an intraperitoneal injection of lipopolysaccharide (LPS, 100 μg/kg) or saline. The magnitude of changes of Tb during the LPS-induced fever was found to be similar between sedentary and trained rats. In sedentary rats, H_2_S production was not affected by LPS. Conversely, in trained rats LPS caused a sharp increase in H_2_S production rate that was accompanied by an increased CBS expression profile, whereas 3-MPST and CSE expressions were kept relatively constant. Sedentary rats showed a significant LPS-induced release of cytokines (IL-1β, IL-6, and TNF-α) which was virtually abolished in the trained animals. Correlation between POA H_2_S and IL-6 as well as TNF-α was observed. Corticosterone levels were augmented after LPS injection in both groups. We found correlations between H_2_S and corticosterone, and corticosterone and IL-1β. These data are consistent with the notion that the responses to systemic inflammation are tightly regulated through adjustments in POA H_2_S production which may play an anti-inflammatory role downmodulating plasma cytokines levels and upregulating corticosterone release.

## Introduction

Hydrogen sulfide (H_2_S), which is traditionally known as a toxic gas, has been documented to be endogenously produced from L-cysteine in a number of mammalian cells including in the brain [1] and to play important roles in several physiological and pathophysiological conditions. In the brain, this gas is mainly generated through the enzyme cystathionine-β-synthase (CBS) and then modulates synaptic activity [2], increases the survival rate of mice exposed to lethal hypoxia [3], and seems to play a pivotal anti-inflammatory role [1], including in the preoptic area of the hypothalamus (POA) [4] the hierarchically most important region involved in thermoregulation [*cf*. 5]. Considering that the POA is crucial to integrate the physiological responses to immune challenges, it seems plausible to hypothesize that preoptic H_2_S modulates the effects of physical exercise on the febrigenic signaling.

It has long been documented the immune benefits of fever [for review see 5]. Fever is a brain-driven regulated increase in deep body temperature (Tb) and a hallmark of the acute phase response of systemic inflammation [5,6]. This thermoregulatory response can be experimentally induced by administration of exogenous or endogenous pyrogens, and is the product of coordination of inflammatory mediators [5]. Lipopolysaccharide (LPS) is widely used to induce fever in animal models of systemic inflammation. This bacterial molecule induces the release of inflammatory mediators such as cytokines: interleukin-1β (IL-1β), interleukin-6 (IL-6) and tumor necrosis factor-α (TNF-α) [7], which are key molecules involved in the febrigenic signaling.

Physical exercise causes structural and functional alterations in various physiological systems, including the immune system [8]. The experimental protocols of the present study provide an integrated analysis where rats (sedentary and exercised), immune-challenged with LPS, had their POA H_2_S production rate measured as well as their protein expressions of H_2_S-related synthases CBS, 3-mercaptopyruvate sulfurtransferase (3-MPST) and cystathionine γ-lyase (CSE) along with plasma levels of cytokines and corticosterone (CORT).

In rats, it has been reported that animals when trained on a treadmill for four weeks show attenuated levels of TNF-α and IL-1β after LPS administration in a sepsis-like condition which differs considerably from the present study since those rats had circulatory shock after 10 mg/kg of LPS given intravenously [9]. Rats may also be voluntarily trained in a wheel. Rowsey and cols. [10,11] measured Tb and cytokines after eight weeks of training in a running wheel and found that trained rats had an exacerbated thermoregulatory response to LPS and had plasma levels of IL-10 and IL-1β increased and decreased, respectively, in comparison to sedentary animals, whereas IL-6 and TNF-α were observed to be unaltered. These studies have not explored the putative integration of the immune responses measured through plasma levels of cytokines and CORT combined with Tb measurements in the same experimental conditions. Needless to say, no previous study has assessed the role of hypothalamic H_2_S in modulating the physiological responses to LPS in trained rats.

Therefore, given that (i) H_2_S has been shown to be endogenously produced in the POA and to modulate the LPS fever, and (ii) physical exercise affects the immune system modulating the production and release of cytokines and CORT, the present study was undertaken to test the hypothesis that the modulatory effect of physical exercise on the febrigenic signaling is regulated by the production of H_2_S in the POA.

## Materials and Methods

### Animals

Seventy five male Wistar rats (140–150 g–at the beginning of physical training program) were obtained from the vivarium of the University of São Paulo, Campus Ribeirão Preto. The rats were group-housed in cages with a metallic grid lid and the floor covered with wood chip bedding material (4 animals per cage), had free access to water and food and were housed in a temperature-controlled chamber at 24–25°C (model: ALE 9902001; Alesco Ltda., Monte Mor, SP, Brazil), with a 12:12-h light:dark cycle (lights on at 6:00 AM). Experiments started between 08:00 and 10:00 AM to prevent effects of circadian variation. This study was carried out according to the Guide for the Care and Use of Laboratory Animals of the National Council for the Control of Animal Experimentation (CONCEA). Experimental protocols were approved by the Local Animal Ethical Committee of the Dental School of Ribeirão Preto, University of São Paulo (2015.1.973.58.7).

### Physical exercise on treadmill

Animals were habituated on a motor-driven treadmill (Insight EP 131, Brazil) and put to run for 5 days to 10, 15, 20, 25, and 30 min/day and the incremental speeds/day was 10, 12, 13, 14, 15 m/min (0° slope). After that, the rats were randomly divided into four groups: sedentary-saline, sedentary-LPS, exercise-saline and exercise-LPS. Exercise groups ran for 4 weeks. Briefly, in the 1st week, the animals ran at 15 m/min for 30 min/day. Then, treadmill speed and exercise duration were progressively increased to 17 m/min for 40 min/day in the 2nd week. During the 3rd week the speed was 19 m/min for 50 min/day and on the 4th week the rats ran at 20 m/min for 60 min/day. This method was adopted from previous studies in rat [12] and in mice [13].

### Surgery

Surgical procedure was performed under ketamine–xylazine anesthesia (100 and 10 mg/kg, respectively; 1 ml/kg, intraperitoneal, i.p.). The rats were submitted to a median laparotomy for insertion of a temperature datalogger capsule (SubCue, Calgary, Alberta, Canada) into the peritoneal cavity. After the surgical procedure, they received a prophylactic dose of antibiotic (160,000 U kg^−1^ benzylpenicillin, intramuscularly) and analgesic medication was also provided (Flunixin; 2.5 mg kg^−1^, subcutaneously). This surgery was performed one week before the experiments.

### Deep body temperature (Tb) measurements

Tb of the animals was recorded at 5-min intervals throughout the experiments by the temperature datalogger capsule.

### Experimental protocols

In our experimental protocols, after the last training session (or not–the sedentary groups), the rats were placed individually in a room with ambient temperature (Ta) set at 24°C; this Ta was maintained constant throughout the experiments. Twenty four hours after the last training session, trained rats received a dose of 100 μg/kg (intraperitoneal, ip) of LPS (serotype 0111:B4, Sigma, St. Louis, MO, USA) dissolved in pyrogen-free saline. Saline was injected into another group of trained rats as control (1 ml/kg, ip). The groups of sedentary rats received the same treatment, *i*.*e*., injection of either LPS or saline at the same dose. The Ta and the LPS dose were chosen based on previous study [4]. During the experimental protocols, rats were monitored continuously as to their behavior. Control rats treated with saline, showed no changes in their behavior, body condition, and well-being. Conversely, rats treated with LPS showed typical signs of illness such as reduced mobility and piloerection, but we observed no mortality.

#### Protocol 1

This experimental protocol was designed to evaluate the effects of physical exercise on euthermia or the LPS-induced fever. LPS (100 μg/kg, ip) or saline (1 ml/kg, ip) was administered to sedentary and trained rats. Tb was measured for 6 h, starting 1 h before the treatments.

#### Protocol 2

The second experimental protocol was aimed at evaluating 2 h after LPS administration the production rate of H_2_S in the POA, and plasma levels of cytokines (IL-1β, IL-6 and TNF-α) and CORT. LPS (100 μg/kg, ip) or saline (1 ml/kg, ip) was injected into sedentary or trained rats. Tb was measured for 3 h, starting 1 h before the treatments. Two hours after the administration of LPS or saline, the rats were decapitated and the blood was collected and processed as described below.

#### Protocol 3

This experimental protocol was aimed at evaluating protein expressions profiles of CBS, 3-MPST and CSE in the POA of rats 2 h after LPS administration.

LPS (100 μg/kg, ip) or saline (1 ml/kg, ip) was injected to sedentary or trained rats.

### AVPO sampling

Sedentary and trained rats were decapitated 2 h after LPS or saline administration and their brains were quickly excised, promptly frozen by submersion in dry ice-cold isopentane, and stored at −70°C. The anteroventral region of the preoptic area (AVPO) of the hypothalamus was sampled in a cryostat by a punch needle (0.9 mm inner diameter) from a 500-μm thick slice for the protocol 2 and 1500-μm thick slice for the protocol 3 of the anterior hypothalamus, based on the following landmarks: ventral, optic chiasm; dorsal, anterior commissure; median, the 3V. Bilateral punches were taken just above the dorsal boundary of the optic chiasm and at the left and right lateral wall of the 3V.

### Measurements of H_2_S production rate in the POA

H_2_S levels were determined as previously described [4,14–17]. AVPO bilateral samples were homogenized in potassium phosphate buffer (100 mM; pH 7.4) using a microprocessor (VirTis, Gardiner, NY, USA). Each sample (50% w/v; 100 μl) contained L-cysteine (10 mM; 20 μl), pyridoxal 5′-phosphate (2 mM; 20 μl) and PBS (30 μl). The reaction was performed in parafilmed eppendorf tubes and initiated by transferring the tubes from ice to bath at 37°C. After incubation for 2 h, zinc acetate (1% w/v; 100 μl) was added to trap evolved H_2_S followed by tricloroacetic acid (10% w/v; 100 μl) to precipitate proteins and thus stop the reaction. After centrifugation, N,N-dimethyl-p-phenylenediamine sulphate (20 mM; 50 μl) in HCl 7.2 M followed by FeCl3 (30 mM; 50 μl) in HCl 1.2 M was then added to 50 μl of the supernatant, and optical density was measured at 670 nm. The calibration curve of absorbance was obtained using Na_2_S solutions (0.1–100 μg/ml). To assess the protein content of the samples, the pellets were diluted in 4 ml of sodium hydroxide (0.1 N). The solution was then assayed by using a protein dye reagent (Bio-Rad Laboratories; Hercules, CA, USA; code number: 500–0006).

### Western blot analysis

Protein expressions of H_2_S-related synthases CBS, 3-MPST and CSE in the AVPO were measured using immunoblotting assay. The tissue from bilateral AVPO was homogenized at 4°C in lysis buffer (containing 50 mM Tris–HCl, pH 7.4, 150 mM NaCl, 1% Triton X-100, 0.1% SDS, 1 μg/mL aprotinin, 1 μg/mL leupeptin, 1 mM phenylmethylsulphonyl fluoride, 1 mM sodium orthovanadate, pH 10, 1 mM sodium pyrophosphate, 25 mM sodium fluoride and 0.001 M EDTA, pH 8). Tissue homogenates were centrifuged at 40.000 rpm for 10 minutes at 4°C and supernatant was collected for analysis. Protein concentration in tissues homogenates was determined by Bradford method, using a protein dye reagent (Bio-Rad Laboratories; Hercules, CA, USA; code number: 500–0006). Aliquots containing 30 μg of protein were dissolved in loading buffer, proteins were separated by sodium dodecyl sulfate polyacrylamide gel electrophoresis (10%), transferred to nitrocellulose membranes, incubated for 1 h in 50 mL of blocking buffer (PBS, 2.5% skim milk) and washed in buffer (PBS, 0.1% Tween 20, pH 7.6). Then, membranes were incubated with the corresponding primary antibody in 5% bovine serum albumin, overnight at 4°C. Primary antibodies included monoclonal rabbit anti-mouse CBS (1:1000; Cell Signaling Technology, Beverly, MA), polyclonal rabbit anti-mouse MPST (1:250; Sigma-Aldrich, St Louis, MO), or monoclonal mouse anti CSE (1:3000; Abnova Corporation, Taipei, Taiwan). Thereafter, membranes were washed and incubated with secondary antibodys horseradish peroxidase-conjugated goat anti-mouse (p-CREB) and anti-rabbit (CBS and p-Akt) (1:5 000; Dako, Glostrup, Denmark) for 1 h at room temperature. Labeled proteins were detected using the Supersignal West Pico Chemiluminescent substrate (Pierce, Rockford, IL, USA). For stripping and reprobing, membranes were recovered in stripping buffer (100 mM 20-mercaptoethanol, 2% sodium dodecyl sulfate, 62.5 mM Tris-HCl, pH 6.8) during 30 minutes at 50°C. To adjust the equivalence of protein loading and/or transfer, membranes were washed with TBS-T before blocking and incubated with monoclonal primary antibody against anti-α1-tubulin (1:5000, Sigma-Aldrich, in 5% bovine serum albumin) overnight, at 4°C. Finally, the intensity of the identified lanes was quantified with image analysis system (Molecular Imaging Systems, Eastman Kodak Company, Rochester, NY). The data were normalized using α1-tubulin and data represented as relative expression (enzyme of interest/ α1-tubulin).

### Measurements of plasma levels of cytokines and CORT

Sedentary and trained rats were decapitated 2 h after LPS or saline administration and the blood was collected in heparin-coated tubes. Afterwards, the tubes were centrifuged (3.500 rpm, 20 min, 4°C) and plasma was stored at −70°C. Plasma levels of IL-1β, IL-6 and TNF-α were determined using specific enzyme-linked immunosorbent assay (ELISA) kits for each cytokine (R&D Systems, Minneapolis, Minn., USA) according to the manufacturer’s instructions. Plasma levels of CORT were measured using a specific radioimmunoassay technique [18]. Plasma corticosterone levels were determined using a specific radioimmunoassay after extraction with ethanol. Corticosterone antiserum was purchased from Sigma, and 1,2,6,7-3 H-corticosterone was from GE Healthcare Life Sciences (Milwaukee, WI, USA). The assay sensitivity and the intra- and inter-assay variability coefficients were 0.4 lg/dl, 5.1% and 8.4%, respectively.

### Statistical analysis

Data are expressed as means ± standard deviation (SD). Tb values (°C) plotted at 5-min intervals are shown as raw values. Initial Tb (Tbi) represents the values of Tb measured at 5-min intervals averaged over the 60 min of the acclimatization period. Thermal index, expressed as °C × min, were calculated from area under curve, from -60 to 0 min (basal records), and from 240 to 300 min (febrile phase). H_2_S levels in the POA are expressed as nmol/mg protein/h. Protein expression profiles are given as relative expression of the enzyme of interest / α1-tubulin. Plasma levels of IL-1β, IL-6, TNF-α and CORT are expressed as pg/mL and ng/mL, respectively. Statistical differences among groups were determined by two-way ANOVA followed by and Bonferroni post hoc test. Pearson’s correlation test was used to determine correlation between variables. The level of significance was set at P<0.05.

## Results

### Effect of physical exercise on the LPS-induced fever

**Fig 1:**
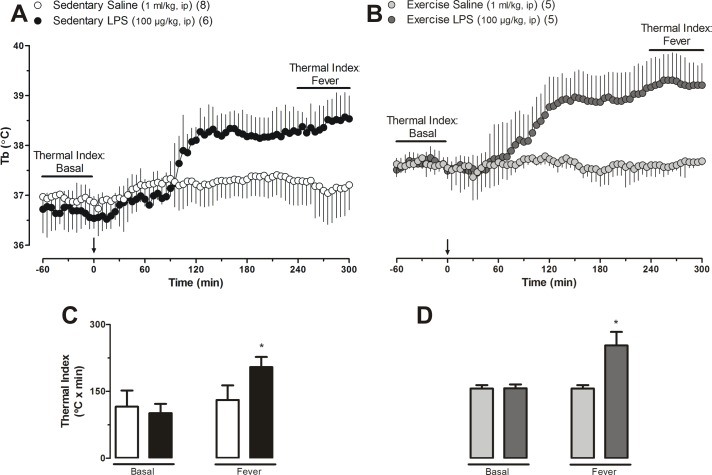
Time courses of deep body temperature (Tb) of sedentary (A) and trained (B) rats showing the effects of systemic LPS administration. Thermal indexes (C and D) of basal and febrile periods clarify the data shown by the horizontal bars in the panels A and B respectively. Arrow indicates the moment of the ip injection of LPS or saline. Values are means ± SD. Number of animals in each group is shown in parenthesis. * P<0.05, Groups treated with LPS vs. the respective control group.

We investigated the effect of physical exercise on the modulation of the LPS-induced fever. Intraperitoneal administration of saline caused no changes in Tb of sedentary and exercised rats. On the other hand, ip injection of LPS caused the typical febrile response in these two groups of rats (Fig 1A and 1B). Thermal indexes of basal and febrile phase (area under curve; indicated by the horizontal bars in the Fig 1A and 1B) were calculated to clarify the changes in Tb observed in sedentary (Fig 1C) and trained rats (Fig 1D). As shown in Fig 1C, LPS caused a significant (P<0.05) increase in Tb (fever) of sedentary rats. Fig 1D shows that administration of LPS to trained rats also evoked a significant (P <0.05) rise in Tb (fever) when compared to the respective control group (i.e., exercise saline). Fig 1 shows that the changes in Tb of trained rats injected with saline (Fig 1A) or LPS (Fig 1B) were not different (P>0.05) from those of sedentary rats injected with saline or LPS, respectively.

### Effect of physical exercise combined or not with LPS on the production rate of H_2_S in the POA

**Fig 2:**
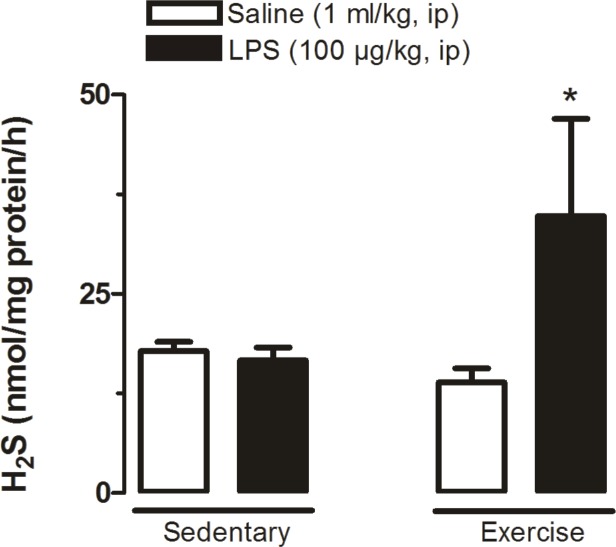
H_2_S levels in the POA at 120 min after systemic administration of LPS or saline. Sedentary saline (n = 8), Sedentary LPS (n = 6), Exercised saline (n = 5) and Exercised LPS (n = 5). * P<0.05, Exercised LPS vs. the other three groups.

Trained rats treated with LPS had significantly (P<0.05) higher levels of H_2_S compared to the other three groups (Fig 2).

### Effect of physical exercise combined or not with LPS on the protein expressions of the CBS, 3-MPST and CSE enzymes in the POA

**Fig 3:**
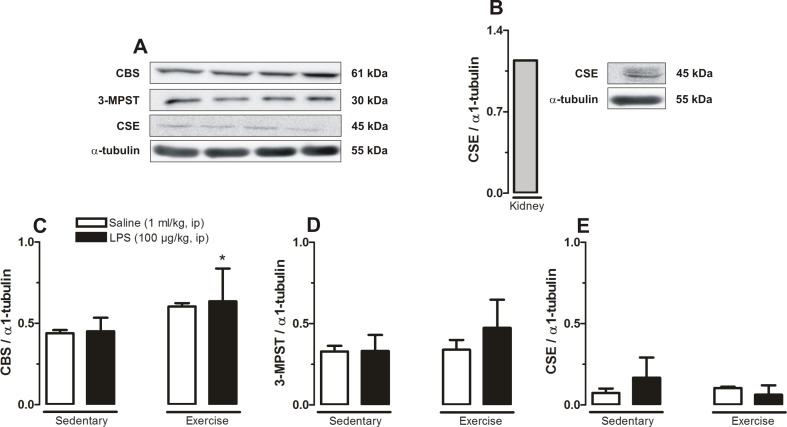
Relative proteins (CBS, 3-MPST and CSE) expression levels in the POA at 120 min after systemic administration of LPS or saline. Painel A shows representative bands reflecting chemiluminescence signal intensity for studied proteins of interest and loading control in each experimental group. Painel B shows the relative expression and band reflecting chemiluminescence signal intensity of CSE and loading control of the rat kidney, used as control. Results are expressed as relative expression of CBS (C), 3-MPST (D) and CSE (E). Sedentary saline (n = 7), Sedentary LPS (n = 7), Exercised saline (n = 6) and Exercised LPS (n = 7). * P<0.05, Exercised LPS vs. Sedentary Saline and Sedentary LPS.

Protein expressions in rat POA were evaluated by western blotting. The CBS expression profile was increased (P<0.05) in trained rats treated with LPS comparing to all other groups. Neither training nor LPS caused any significant difference in both 3-MPST and CSE expressions profiles (Fig 3). Kidney tissue (panel B) was used as an additional control.

### Effect of physical exercise combined or not with LPS on plasma levels of IL-1β, IL-6 and TNF-α

**Fig 4:**
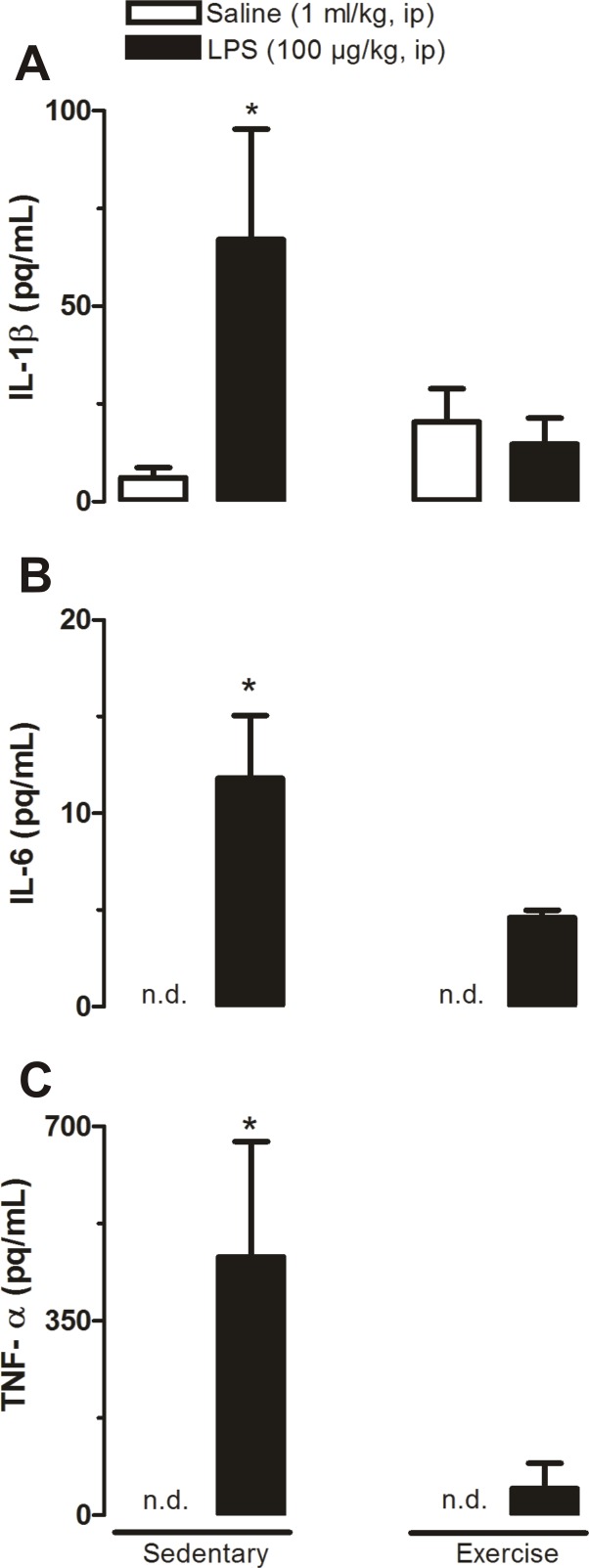
**Plasma levels of IL-1β (A), IL-6 (B) and TNF-α (C) at 120 min after systemic administration of LPS or saline.** Sedentary saline (n = 8), Sedentary LPS (n = 6), Exercised saline (n = 5) and Exercised LPS (n = 5). * P<0.05, Sedentary LPS vs. the other three groups.

To investigate the effects of physical exercise on the febrigenic signaling, we assessed plasma levels of cytokines (IL-1β, IL-6 and TNF-α) in trained rats injected with LPS. Sedentary rats were used as control. We found statistical difference (P<0.05) comparing the group Sedentary LPS with the other three groups (Fig 4).

### Correlation between POA H_2_S and plasma levels of cytokines

**Fig 5:**
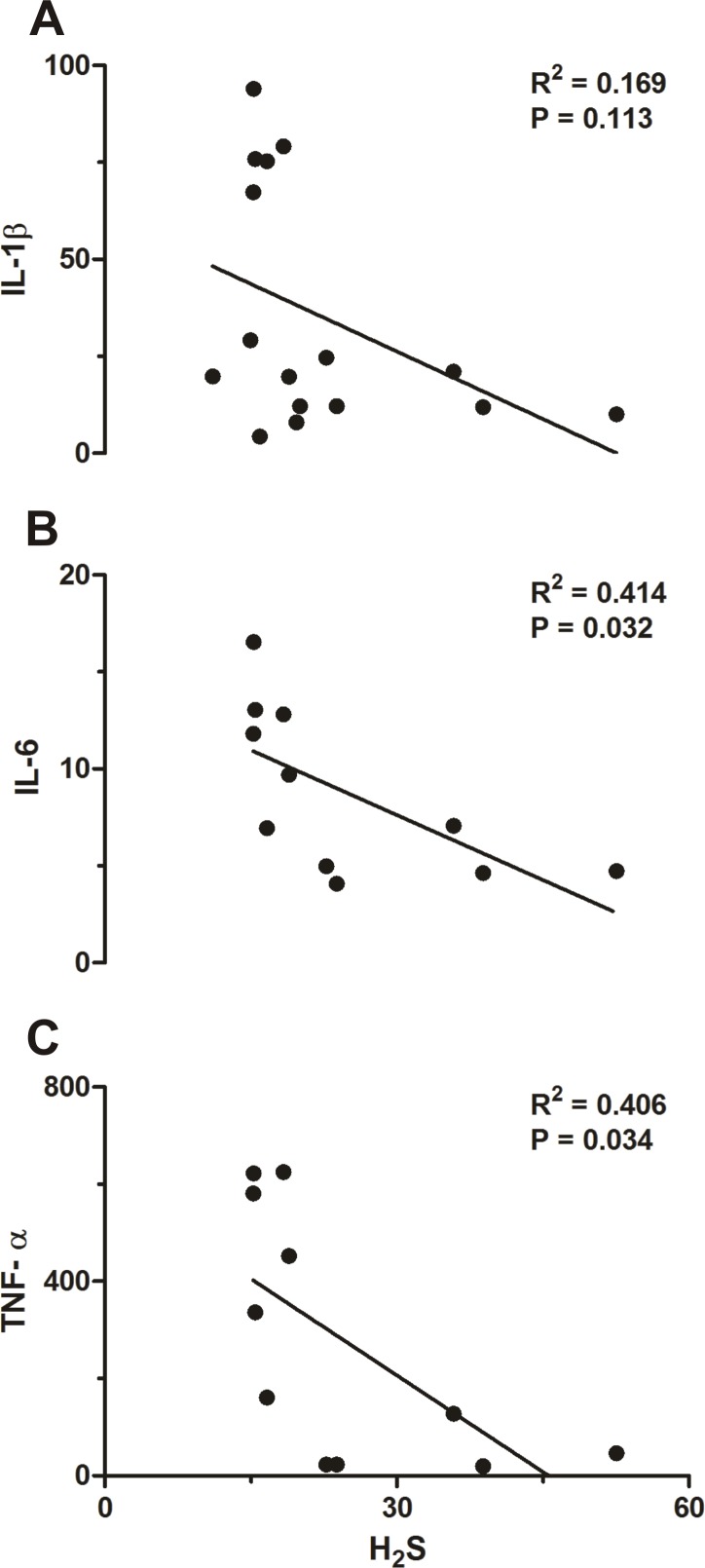
**Correlation analyses between H_2_S and IL-1β (A), IL-6 (B) and TNF-α (C)**.

Fig 5 shows correlations between H_2_S production rate in the POA and plasma levels of IL-6 (R = 0.414, P = 0.032) and TNF-α (R = 0.406, P = 0.034; Fig 5B and 5C) were observed. No correlation was found between H_2_S and IL-1β (R = 0.169, P = 0.113; Fig 5A).

### Effect of physical exercise combined or not with LPS on plasma levels of CORT

**Fig 6:**
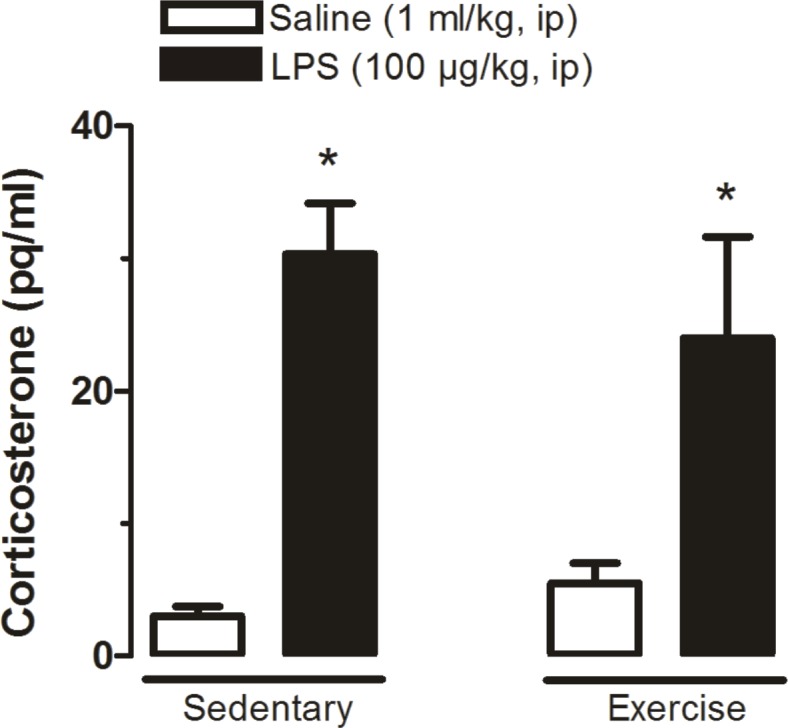
Plasma corticosterone levels at 120 min after systemic administration of LPS or saline. Sedentary saline (n = 8), Sedentary LPS (n = 6), Exercised saline (n = 5) and Exercised LPS (n = 5). * P<0.05, Groups treated with LPS vs. the respective control group.

Sedentary and trained rats injected with saline did not present significant levels of CORT. The groups treated with LPS had significantly (P<0.05) higher levels of CORT compared to the groups treated with saline. No statistical differences (P>0.05) were found between the groups Sedentary LPS and Trained LPS (Fig 6).

### Correlation between POA H_2_S and plasma levels of CORT

**Fig 7:**
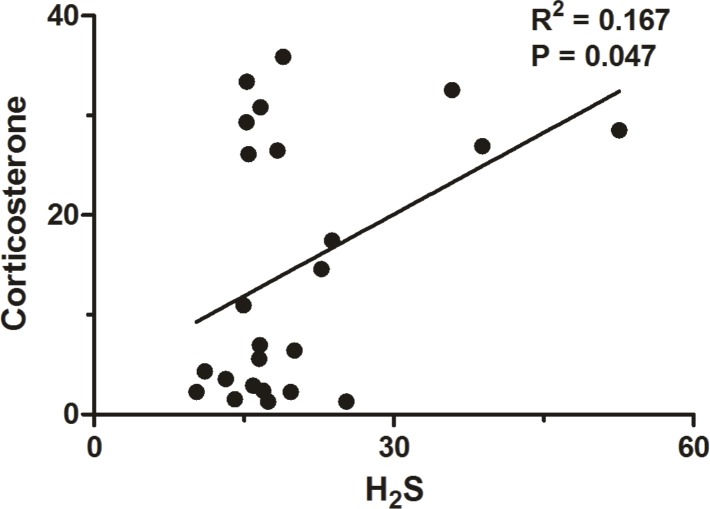
Correlation analysis between H_2_S and corticosterone.

A correlation between H_2_S production rate in the POA and plasma levels of CORT (R = 0.167, P = 0.047; Fig 7) was observed.

### Correlation between plasma levels of CORT and cytokines

**Fig 8:**
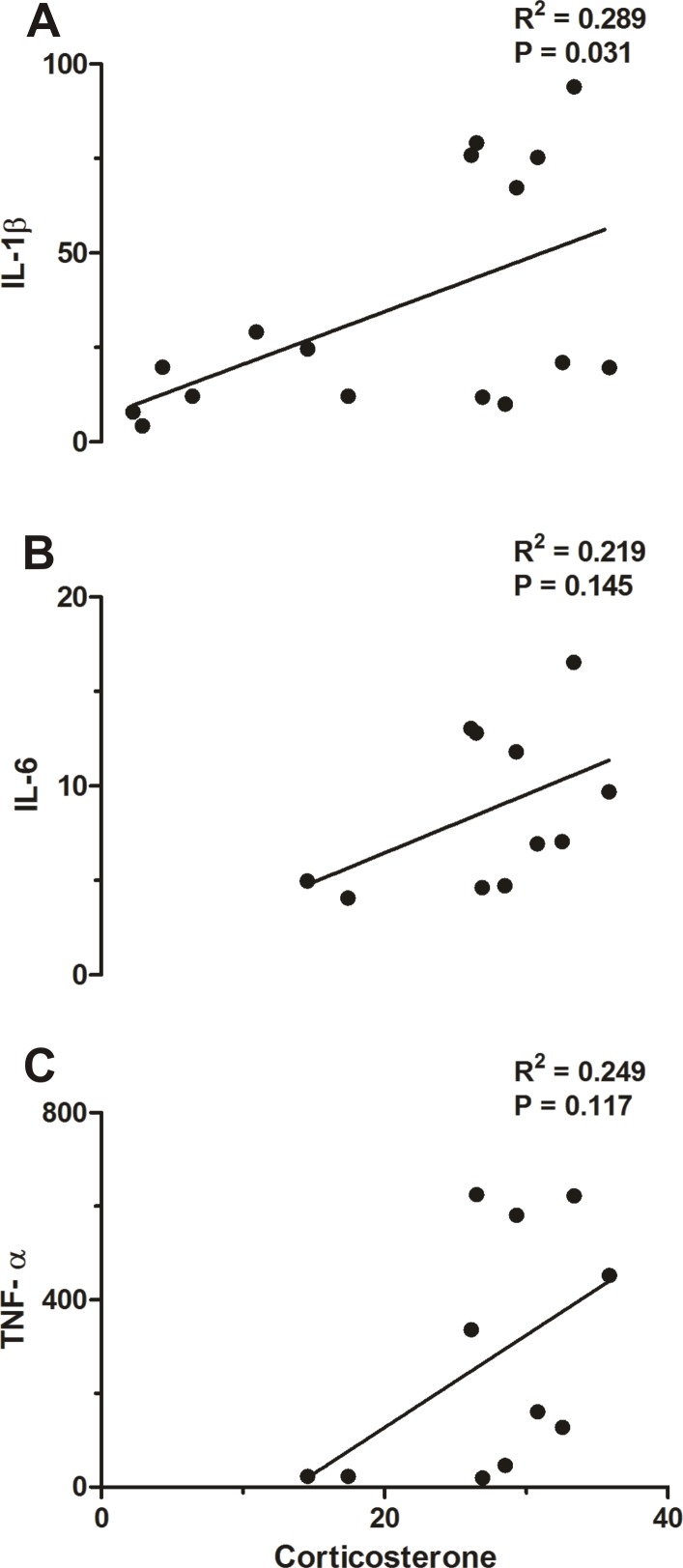
**Correlation analyses between corticosterone and IL-1β (A), IL-6 (B) and TNF-α (C)**.

A correlation between plasma CORT and IL-1β (R = 0.289, P = 0.031; Fig 8A), but not between CORT and IL-6 (R = 0.219, P = 0.145; Fig 8B) and TNF-α (R = 0.249, P = 0.117; Fig 8C) was observed.

## Discussion

In the present study, we investigated the effects of physical exercise on the POA production rate of H_2_S along with the protein expressions of H_2_S-related synthases CBS, 3-MPST and CSE in this region and its relation to the febrigenic signaling during systemic inflammation. Interestingly, the immune challenge caused a sharp increase in POA H_2_S and CBS expression in trained but not in sedentary rats. This exacerbated response of trained rats contrasts with a blunted LPS-induced plasma cytokines release (IL-1β, IL-6 and TNF-α; Fig 4). Indeed, correlation analyses revealed that the POA H_2_S production rate is inversely proportional to cytokines plasma levels, specifically IL-6 and TNF-α (Fig 5).

As to limitations of the present study: (i) here we speculate that the enhanced levels of H_2_S (Fig 2) observed in trained rats treated with LPS are related to reduced plasma cytokines levels, based on a correlation analysis. However, our data does not provide a background for a causal relationship to the drop in LPS-induced increase of cytokines plasma levels of trained rats, because we did not inhibit H_2_S production in the POA; and (ii) correlations with the expressions profiles of the enzymes CBS, 3-MPST and CSE was precluded because different sets of rats were used.

Originally, we had thought that LPS-induced fever in trained rats would be attenuated. Interestingly, our findings do not support such assumption given that the magnitude of the LPS-induced changes in Tb of trained versus sedentary rats was found to be similar (Fig 1). In other words, physical exercise does not alter the magnitude of the febrile response to LPS in trained rats compared to sedentary ones. At first glance it would seem that fever would be potentiated by physical exercise (Fig 1A and 1B), as the highest values of Tb (at 300 min after LPS) of trained rats are considerably higher than those of the sedentary ones. However, this may be explained by the fact that the basal euthermic values of Tb of the trained rats are higher. This finding is in agreement with that by Rowsey and cols. [19] who reported that physical exercise is associated with a higher Tb at rest. This increased basal Tb during euthermia has been attributed to the increase in heat production [10].

The gas H_2_S is ‘classically’ known as a poison that smells like rotten eggs. Since this gas was originally considered as a toxic gas only, its beneficial cytoprotective actions were ignored until it was described as a neuromodulator, in the mammalian brain. H_2_S is endogenously produced from l-cysteine by the enzymes CBS, 3-MPST and CSE. The relative contribution of each one of these enzymes in producing H_2_S centrally is different. For instance, the levels of CSE in the brain have been reported to be particularly low and consequently its contribution to the neuronal activity are thought to be negligible, in relation to CBS (the main isoform in the CNS) and 3MST [20,21], which is in agreement with our data (Fig 3).

It is well established that LPS stimulates peripheral release of cytokines that ultimately trigger a brain-driven elevation of Tb (fever) [5]. The effects of physical exercise on the immune system have long been studied but it remains not fully understood. It has been proposed that physical exercise may be beneficial or harmful to the body’s defense system [22,23]. Our results clearly show that physical exercise may affect immune responses to endotoxemia since we found a reduced LPS-induced release of cytokines in trained rats, which is consistent with the notion that physical training considerably decreases the inflammatory signaling (Fig 4).

In an acute session of physical training, it has been documented increased levels of cytokines at the end of the training, returning to basal values within a few hours [22]. In the present study, in which rats were submitted to chronic physical training and plasma samples were obtained 24 h after the last training session, we found that plasma levels of cytokines were not affected by physical exercise per se (Fig 4), which is in agreement with previous studies in running [24] and swimming rats [25].

Cytokines such as IL-1β, IL-6 and TNF-α are considered to be key mediators of the febrigenic signaling. They are synthesized by activated macrophages and monocytes in response to LPS [26,27]. In the present study, we showed that physical exercise does attenuate the LPS-induced release of IL-1β, IL-6 and TNF-α (Fig 4).

Specifically, plasma IL-1β levels were observed to be kept unaltered in trained rats treated with saline whereas LPS caused about a 15-fold increase in this cytokine. This major LPS-induced IL-1β increase was completely prevented by physical training. Our data differ from a previous report that showed that IL-1β levels are increased in rats that voluntarily exercise on running wheels compared to sedentary rats [11]. However, a similar tendency (even though not significant) was observed in the present study (Fig 4A). As to the effect of LPS, it has been reported an attenuated IL-1β levels during endotoxic shock in trained rats [9], which is in agreement with the present study (Fig 4A). As to other cytokines, Harden et al. [28] also using voluntary wheel running observed that exercise does not result in blunting in the levels of plasma cytokines induced by LPS at the dose of 250μg/kg. Therefore, it seems plausible to believe that not only LPS dose, but also the experimental design need to be taken under consideration when reconciling the available data.

Plasma levels of the other cytokines, *i*.*e*., IL-6 and TNF-α were not affected by physical exercise in euthermic rats treated with saline. Conversely, during endotoxemia these cytokines were observed to be significantly increased and similarly to IL-1β, physical exercise blunted these LPS-induced increased plasma levels (Fig 4B and 4C). Interestingly, IL-6 neutralization in rats [7] or its absence in knockout mice [27] causes an attenuated LPS fever. IL-6 release in the blood may be due to the action of IL-1β [7,29], synthesized immediately after the LPS-induced febrile response in peripheral tissues and the brain, mediating fever [29,30].

Therefore, it is not surprising that a similar pattern of responses of IL-1β and IL-6 were observed in the present study. It is clear-cut that TNF-α plays a role in immune response. However, its participation may differ among experimental designs. According to this notion, Bagby and cols. [31] showed that after a single exercise session combined with a LPS injection, trained rats were observed to have lower TNF-α levels compared to those of sedentary ones. Conversely, 12-week exercise training has been shown to cause increased plasma TNF-α levels in response to LPS injection [32]. These findings are in disagreement with the present study since we found an attenuated effect of LPS on TNF-α by chronic physical exercise (Fig 4C). This discrepancy may be related to the different experimental designs used in the studies since both previous reports [31, 32] injected LPS at higher doses than that one of the present study.

However, these discrepancies mentioned above may not explain a particular set of data related to the levels of plasma IL-1 that was found to be higher than those of IL-6 (Fig 4). Our findings are in disagreement with a number of studies [28,33] that showed that levels of IL-6 are much higher than those of IL-1 after stimulation with LPS. Moreover, discrepancies also exist in relation to measurable base-line concentrations of cytokines during basal conditions. While we and others [33,34] found IL-6 is detectable under control conditions, this same cytokine could be detected in some studies [11,24]. To the best of our knowledge, we are afraid the explanation to these discrepancies remains rather unclear.

Another important finding of the present study is that physical exercise did not alter the immune activation of the hypothalamic–pituitary–adrenal (HPA) axis (Fig 6). Different types of exercise (voluntary or forced, for instance) may have different impacts on physiological systems including the endocrine system [35,36]. In agreement with this notion, physical exercise activates the HPA axis resulting in changes in CORT immediately after exercise, and plasma CORT levels are known to affect Tb [37]. Interestingly, in the present study, physical exercise did not cause any significant changes in plasma CORT levels in saline-treated rats (Fig 6), 24 hours after the last exercise session.

Yet, another reason to assess CORT is based on the fact that this hormone may be used as an indicator of stress [38]. Interestingly, we observed that LPS treatment caused a sharp increase in plasma CORT levels, and that this effect was similar in both sedentary and trained rats, indicating that CORT may have affected the LPS-induced fever similarly in the two groups (Figs 1 and 6).

The present study adds the gaseous molecule H_2_S endogenously produced in the POA as a key modulator not only of cytokines release but also of the HPA axis. In the central nervous system, H_2_S is synthetized by the enzyme CBS, mainly in astrocytes [39,40]. Kimura et al. [41] reported a reciprocal interaction between neurons and astrocytes, which is also true for cells in the POA that account for the control of fever [5,42]. To our knowledge, the present study is the first to report an important role of POA H_2_S in blunting the acute-phase reaction of systemic inflammation in trained rats.

In sedentary rats, it has been previously shown that H_2_S acts as an antipyretic molecule in the brain and that LPS fever is accompanied by reduced levels of POA H_2_S production rate [4]. The present study corroborates with these findings (Fig 2) and adds experimental evidence by showing that in trained rats treated with LPS the POA H_2_S production rate is actually drastically increased (Fig 2) rather than attenuated. Interestingly, here we also showed that the action of H_2_S as an antipyretic molecule seems to be at least in part mediated by an important suppression of plasma cytokines (Fig 5) besides a stimulation of HPA axis (Fig 7). Actually, our results are consistent with the notion that POA H_2_S production has a positive relation to the HPA axis, based on the data shown in Fig 7. However, limited by our experimental design, we cannot infer if this is a modulatory effect of the HPA axis over POA H_2_S production or otherwise, *i*.*e*., a modulatory effect of POA H_2_S production over the HPA axis. As to the latter possibility, Dello Russo et al. [43] tested the effect of H_2_S on the release of corticotropin-releasing hormone (CRH) from rat hypothalamic explants, and found that an H_2_S donor (NaHS) had no effect on CRH basal secretion but decreases the KCl-stimulated CRH release. Perhaps, avoiding the severe potential toxic effect of the gas donor, they also increased the endogenous H_2_S production using an indirect precursor of H_2_S formation (S-adenosyl-lmethionine—SAMe), which similarly to the results obtained with the donor had no effect on HPA function under resting conditions, but inhibited stress-related glucocorticoid increase. Even though, one may argue that reconciling data obtained from studies that used different stimuli (stress and LPS challenge) and that may have had a possible toxic effect of the gas donor, in essence their data are in disagreement with our results shown in Fig 7. However, it is worth mentioning that in Dello Russo’s [43] study, SAMe has been used as a pharmacological tool in relatively high doses because of its poor diffusion, and that SAMe is also endogenously produced in the hypothalamus, where its gene expression and protein synthesis have been shown to be up-regulated by glucocorticosteroids [44] which is in agreement with our data (Fig 7). Furthermore, using a different experimental approach involving a peripheral tissue, a recent study [45] examined the role of CBS and CSE expressed in adrenal glands in the maintenance of mitochondrial function and glucocorticoids production, and found that CBS or CSE inhibitors cause mitochondrial oxidative stress and dysfunction resulting in a blunted corticosterone release in response to adrenocorticotropic hormone. Moreover, they also observed that these effects could be attenuated by an H_2_S donor (GYY4137). Even though obtained by different approaches, data observed by Wang et al [45] corroborate our results (Fig 7). We are afraid further studies are needed before a clearer scenario is settled.

Both the immune stimulus with LPS and the proinflammatory cytokine IL-1β are known to induce a suite of brain-mediated responses, including activation of the HPA axis [46]. Therefore, it is not surprising that we observed a correlation between IL-1β and CORT (Fig 8A).

In summary, physical exercise has been accepted as a major strategy to improve public health. The present study addressed how physical exercise interacts with the immune system by assessing fever (the hallmark of infection), the preoptic production rate of H_2_S, the protein expression of the H_2_S-related enzymes CBS, 3-MPST and CSE, and plasma levels of cytokines and CORT. Physical exercise increased basal Tb during euthermia of trained rats. Interestingly, the LPS-induced fever seemed to be increased in trained rats, but a closer analysis (evaluating the changes in Tb instead of absolute values) revealed that actually the febrile response to LPS is similar between the groups, as also observed for plasma CORT levels (Fig 6). Our data indicate that this maintained thermoregulatory response in both groups is indeed tightly regulated by H_2_S in the POA which is inversely proportional to plasma cytokine levels (particularly IL-6 and TNF-α—Fig 5B and 5C), and directly proportional to the HPA axis activation (Fig 7) which seems to be related to IL-1β plasma concentration (Fig 8).

## Perspectives

To understand the mechanisms underlying the modulatory effect of physical exercise during systemic inflammation, we adopted an experimental design that allows integrated analyses of the interaction among the nervous, endocrine and immune systems.

Physical inactivity leads to loss of muscle mass and increased intra-abdominal fat deposition. Such intra-abdominal adiposity stimulates macrophage infiltration causing the activation of inflammatory cascade reactions, ultimately resulting in chronic systemic inflammation. In this scenario insulin resistance, atherosclerosis, and tumor growth may take place, making individuals prone to develop type 2 diabetes mellitus, cardiovascular diseases and cancer. Conversely, physical exercise stimulates skeletal muscle hypertrophy, improves fat oxidation, increases insulin sensitivity, and causes important anti-inflammatory actions [47].

It should be kept in mind that these anti-inflammatory actions are so finely adjusted during systemic inflammation that the changes in Tb (febrile response) of sedentary and trained rats are virtually identical, and this adjustment seems to be at least in part mediated by POA H_2_S.

## Supporting Information

S1 File Arrive Checklist. (PDF)

S2 File Data. (ZIP)
